# Cytokines IL-6, IL-10, and CCL5 Secreted by Infiltrating B Cells Promote Cell Migration of Human Prostate Cancer Cell Lines

**DOI:** 10.32604/or.2025.073532

**Published:** 2026-03-23

**Authors:** Crystal J. Byrd, Monasia Evans, Woojung Kim, Quintera Knight, Geou-Yarh Liou

**Affiliations:** 1 Department of Biological Sciences, Clark Atlanta University, Atlanta, GA 30314, USA; 2 Center for Cancer Research & Therapeutic Development, Clark Atlanta University, Atlanta, GA 30314, USA; 3 Department of Biological Sciences, Spelman College, Atlanta, GA 30314, USA

**Keywords:** Cytokines, B cells, human prostate cancer cells, cell migration, tumor microenvironment (TME)

## Abstract

**Objective:**

The progression of prostate cancer cells to metastasis is supported by their tumor microenvironment. Within this microenvironment, infiltrating immune cells, such as B cells, can be either anti-tumorigenic or pro-tumorigenic. Our preliminary data showed that a higher density of the infiltrating B cells was found near prostate cancer cells in human cancer tissues, as compared to the benign prostate tissue regions, thus suggesting that infiltrating B cells would promote the progression of prostate cancer cells. In this study, we aim to investigate the role of infiltrating B cells in enhancing the migratory ability of human prostate cancer cells.

**Methods:**

We utilized Transwell^®^ assays to evaluate the migratory ability of human prostate cancer cells in the presence or absence of B cells, B cell-secreted cytokines, and neutralizing antibodies of B cell-secreted cytokines. We also used Western blot and immunofluorescence staining to evaluate the effects of epithelial-mesenchymal transition on the human prostate cancer cells in response to the B cell cytokines.

**Results:**

Our findings showed an increase in migration of human prostate cancer cells in response to co-cultured B cells as well as the identified B cell cytokines: IL-6, IL-10, and CCL5. Neutralization of these cytokines through their specific neutralizing antibodies decreased B cell-induced prostate cancer cell migration. Results from Western blot and immunocytochemistry showed an increase in expression of N-cadherin and Slug, as well as disorganization of ZO-1, amongst the LNCaP cells treated with B cell cytokines.

**Conclusion:**

These results revealed that infiltrating B cells through their secretion factors enhanced prostate cancer cell migratory ability, which may lead to metastasis.

## 1 Introduction

Prostate cancer is one of the leading causes of cancer death in senior men in America [1–4]. With early detection and treatment, this cancer can be properly controlled or removed [5,6]. However, once the cancer metastasizes, many treatments such as radiation and hormone therapy can become less effective. These treatments poorly affect cancer patients’ quality of life because of their severe side effects [7,8]. Innovative treatments that address the progression of prostate cancer at a microenvironment level are needed to provide more non-invasive approaches during the early stages of prostate cancer to minimize cancer progression.

The interaction of cells within the tumor microenvironment (TME) plays a crucial role in cancer progression. Immune cells [4], such as B cells [9–14], are recruited into this microenvironment by cancer cells [15]. As part of the adaptive immune system, B cells play a critical role in the body’s immune response. These cells can produce antibodies that label foreign substances in the body to be eliminated by other antigen-presenting cells, leading to immune responses throughout the body [16–19]. However, when B cells infiltrate into the TME, studies have shown that B cells can contribute to dual responses: tumor-promoting [20] or tumor-killing in relation to tumor progression [17,21–23].

Cell communication within the TME between cancer cells and B cells occurs through secretion factors, such as cytokines and chemokines [11,24–27]. These secretion factors have been shown to play a role in cancer progression, as they activate different receptors that may lead to cancer metastasis and tumor growth [6,28,29]. During an immune response, secretion factors lead to pro-inflammatory or anti-inflammatory responses. Studies have shown with different types of cancers that there could be a correlation between the expression of certain secretion factors in the patients’ serum and their overall prognosis [2,30–32]. Understanding the effects of various secretion factors on prostate cancer progression is fundamental in creating new approaches to novel treatments.

Cancer progression is measured by the ability of cancer cells to progress to the next stage, including spread or metastasis in the body, as the final stage of the disease. Metastasis requires cancer cells to break off cell-to-cell adhesion and cell junctions, a process known as epithelial-mesenchymal transition (EMT) [33,34]. In this study, we investigated how infiltrating B cells promote cell migration of LNCaP cells through their secretion factors. We aimed to identify the underlying mechanisms that lead to the increased cell migration of human prostate cancer LNCaP cells.

## 2 Materials and Methods

### 2.1 Antibodies and Reagents

The N-Cadherin antibody (Cat. # 333900) and ZO-1 antibody (Cat. # 61-7300) were purchased from Invitrogen (Carlsbad, California, USA). Slug antibody (Cat. # 9585) and Histone-H3 (Cat. # 9715) were purchased from Cell Signaling Technology (Beverly, Massachusetts, USA). GAPDH antibody (Cat. # SC-166574) was purchased from Santa Cruz Biotechnology (Dallas, Texas, USA). Human recombinant proteins, including CCL5 (Cat. #300-06), IL-6 (Cat. #AF20006), and IL-10 (Cat. # AE20010), were purchased from PeproTech (Cranbury, New Jersey, USA). The neutralizing antibodies (NAbs) for CCL5 (Cat. # MAB678100), IL-6 (Cat. # MAB2061100), or IL-10 (Cat. MAB217500) were purchased from R&D Systems (Minneapolis, Minnesota, USA), and IgG1 isotype antibody (Cat. # 400102) as the isotype control antibody was purchased from BioLegend (San Diego, California, USA). Other reagents used are indicated within individual sections.

### 2.2 Cell Culture, Co-Culture System, and Cell Treatment

All cell culture media and supplements are from Thermo Fisher Scientific (Waltham, Massachusetts, USA) unless mentioned otherwise. The human prostate cancer cell line LNCaP (Cat. #CRL-1740, American Type Culture Collection [ATCC], Manassas, Virginia, USA) was cultured in RPMI-1640 media with Glutamax Supplemented (Cat. # 61870036) with 10% fetal bovine serum (FBS) (Cat. # A5209401) and 100 U/mL penicillin-streptomycin (P/S) (Cat. # 15140122). Human prostate cancer cell line Du145 (Cat. # HTB-81) from ATCC was cultured in DMEM media (Cat. # SH3024301, Cytiva, Marlborough, Massachusetts, USA) supplemented with 10% FBS (Cat. # A5209401) and 100 U/mL P/S (Cat. # 15140122). The human B-lymphocyte cell line (BC-3) (Cat. # CRL-3615) from ATCC was cultured in RPMI-1640 ATCC modified media (Cat. # A10491-01) supplemented with 20% FBS (Cat. # A5209401) and 100 U/mL P/S (Cat. # 15140122). All cell lines were cultured in a humidified 37°C incubator with 5% carbon dioxide. All cell lines were tested for mycoplasma contamination with Myco-Strip kit (Cat. # rep-mys-10, InvivoGen, San Diego, California, USA) following the manufacturer’s instructions and they were mycoplasma free.

The co-cultured system of prostate cancer (LNCaP [Cat. #CRL-1740] or Du145 [Cat. # HTB-81]) cells and B cells (BC-3, Cat. # CRL-3615) which is referred to using the Transwell assay, both cells were cultured in growth media composed of RPMI-1640 ATCC modified media (Cat. # A10491-01) supplemented with 20% FBS (Cat. # A5209401) and 100 U/mL P/S (Cat. # 15140122) in a transwell chamber with a pore size of 8.0 µm. See Section 2.3 Transwell Assay for details.

Prostate cancer cells and B cells (Cat. # CRL-3615) were treated with neutralizing antibodies, including IL-6 NAb (Cat. # MAB2061100) or CCL5 NAb (Cat. # MAB678100) at a concentration of 5 µg/mL and IL-10 NAb (Cat. #103295-388) at 10 µg/mL. LNCaP (Cat. #CRL-1740) cells were treated with recombinant cytokine protein (CCL5 [Cat. #300-06], IL-6 [Cat. #AE20006], or IL-10 [Cat. # AE20010]) at a concentration of 50 ng/mL of each. For mitomycin C treatment, 1 µg/mL of mitomycin C (Cat. # BP253112,) was used to inhibit cell proliferation without causing cell death in prostate cancer cells as well as in B cells.

### 2.3 Transwell Assay

Cell migration was performed using 24-well Corning Transwell^®^ (Cat. # 3464, Corning, Corning, New York, USA) cell culture chambers that have membranes with a pore size of 8.0 µm. For recombinant cytokines-induced prostate cancer cell migration, or neutralizing antibody treatment, or mitomycin C (Cat. # BP253112) treatment for evaluating B cells-induced prostate cancer cell migration, see Section 2.2 for the used doses. In brief, LNCaP (Cat. #CRL-1740), with a cell density of 5 × 10^3^ or Du145(Cat. # HTB-81), with the cell density of 2.5 × 10^3^, cells suspended in growth media, RPMI-1640 ATCC modified media (Cat. # A10491-01) supplemented with 20% FBS (Cat. # A5209401) and 100 U/mL P/S (Cat. # 15140122), were seeded on the top of inserts, and B cells (Cat. # CRL-3615) with the cell density of 3.5 × 10^5^ were cultured in the bottom chamber. Treatments including recombinant proteins, neutralizing antibodies or mitomycin C (Cat. # BP253112) were added to the bottom chamber. The Transwell plate was returned to the cell incubator immediately for the indicated time: 22 h for LNCaP mitomycin C (Cat. #CRL-1740) cells and 6 h for Du145 (Cat. # HTB-81) cells to migrate. The migrated human prostate cancer cells were fixed with 4% paraformaldehyde (Cat. # 21-010-263, Thermo Fisher Scientific), stained with Wright-Giemsa (Cat. # 95057-514, Azer Scientific, Morgantown, Pennsylvania, USA), and washed with ddH_2_O before imaging. Images of the migrated prostate cancer cells located at the bottom of the insert of the Boyden Chamber were taken using the EVOS XL Core microscope (Cat. # AMEX1100, Thermo Fisher Scientific) with a 10× objective lens. Five randomly selected fields were used for each condition, and the migrated cell numbers were counted using the ImageJ software (ImageJ 1.52a, Wayne Rasband, NIH, Bethesda, MD, USA).

### 2.4 Immunofluorescence Staining

LNCaP (Cat. #CRL-1740) cells cultured in RPMI complete media were treated with or without all 3 recombinant proteins (CCL5 (Cat. #300-06), IL-6 (Cat. #AF20006), and IL-10 (Cat. # AF20010) at the concentration indicated in Section 2.2, for 22 h. Cells were fixed in 4% paraformaldehyde (Cat. # 21-010-263) for 20 min, permeabilized with 0.5% Triton X-100 in PBS for 10 min, and blocked in 10% goat serum (Cat. # 16-210-064, Thermo Fisher Scientific) for 30 min at room temperature. Primary antibodies, including ZO-1 antibody (1:25, Cat. # 61-7300), or N-cadherin antibody (1:25, Cat. # 333900) were then added to samples overnight at 4°C. After TBST (50 mM Tris.HCl [pH 7.6], 150 mM NaCl, 0.05% Tween 20) washes, samples were incubated for 30 min at room temperature with corresponding Alexa Fluor-conjugated secondary antibodies (1:500, Alexa Fluor 488: Cat. # AB150065, Abcam, Cambridge, United Kingdom and Alexa Fluor 594: Cat. # AB150112, Abcam) and DAPI (D1306, Thermo Fisher Scientific), according to the manufacturer’s instructions. Images were taken using an EVOS M5000 microscope (Cat. #A65468, Thermo Fisher Scientific) with a 10× objective lens.

### 2.5 Cell Lysates Collection and Western Blot

Cells were rinsed twice with PBS, lysed in buffer A (50 mM Tris/HCl [pH 7.4], 1% TritonX-100, 150 mM NaCl, and 5 mM EDTA [pH 7.4]), supplemented with a protease inhibitor cocktail (Cat. # AAJ61473XF, Thermo Fisher Scientific), vortexed with the maximal speed for 1 min, incubated on ice for 10 min, and centrifuged at 4°C, 6010× *g* for 5 min and then 18,782× *g* for 30 min. The supernatants were denatured, followed by 8%–12% SDS-PAGE. Resolved proteins were transferred onto nitrocellulose membranes, followed by blocking in 5% Bovine Serum Albumin (BSA) (Cat. # A23016, Thermo Fisher Scientific) in TBST for 30 min at room temperature and incubated with the primary antibodies (N-Cadherin (1:1000, Cat. # 333900), Slug (1:1000, Cat. # 9585), Histone-H3 (1:1000, Cat. # 9715), or GAPDH (1:1000, Cat. # SC-166574) of interest in 5% BSA (Cat. # 50-000-82565, Thermo Fisher Scientific) in TBST overnight at 4°C. The anti-mouse horseradish peroxidase-linked goat antibody (Cat. # 50195917, Thermo Fisher Scientific) or anti-rabbit HRP-linked goat antibody (Cat. # 50195914, Thermo Fisher Scientific) secondary antibodies (1:3000) were added to the membranes for 30 min at room temperature. Images were captured and visualized with ECL (Pierce™ ECL Western Blotting Substrate, Cat. # PI32106, Thermo Fisher Scientific) and X-ray films (Thermo Scientific™ CL-XPosure™ Film, Cat. # PI34091, Thermo Fisher Scientific).

### 2.6 Statistical Analysis

Statistical analysis was conducted by Student’s *t*-test, one-way analysis of variance (ANOVA), and Tukey’s multiple comparisons test using Prism Version 10 (GraphPad Software Inc., San Diego, CA, USA). Data with a *p*-value of less than 0.05 was statistically significant.

## 3 Results

### 3.1 Elevated Migration of Prostate Cancer Cells When Co-Cultured with B Cells

**Figure 1:**
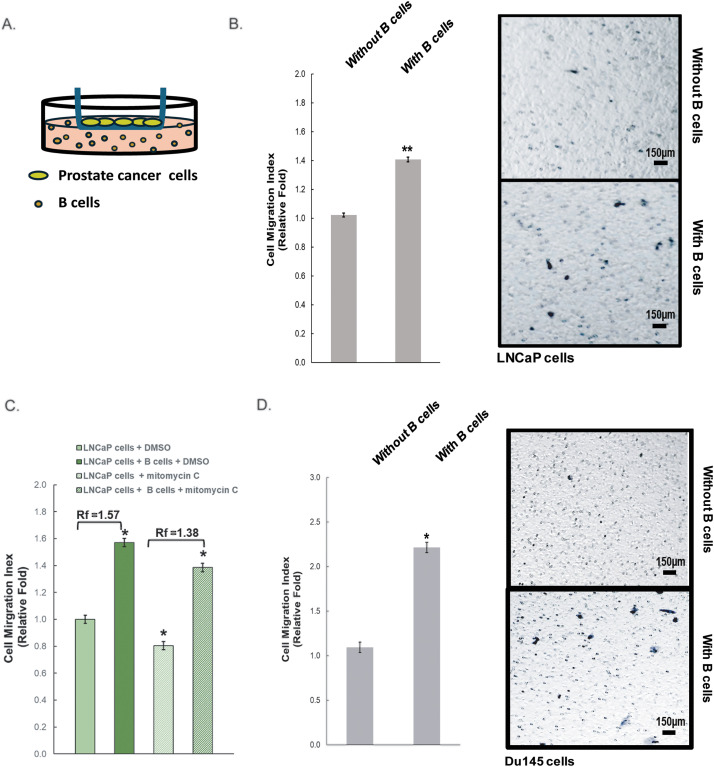
Increased cell migration of prostate cancer cells cultured with B cells. (**A**) Diagram of the Transwell^®^ assay where prostate cancer cells (LNCaP and DU145 cell lines) were in the insert and B cells were in the bottom, separated by a porous membrane. (**B**,**C**) The migration of LNCaP induced by B cells with and without mitomycin C/MMC from (**A**) was quantified. Rf: relative fold change. (**D**) Du145 cells from (**A**) in the presence or absence of B cells were quantified and graphed. Figure shown is representative of three independent experiments. Scale bar: 150 µm. *: *p* < 0.05, **: *p* < 0.01 as compared to the control (without B cells)

Our focus in this study was to investigate the role of infiltrating B cells in the microenvironment of prostate cancer, and their ability to advance early-stage prostate cancer cells to eventually become metastatic. Metastatic cancer possesses elevated cell migratory and invasion abilities. We used the Transwell^®^ chamber assay to examine the effect of infiltrating B cells on prostate cancer cell migration, as shown in Fig. 1A. LNCaP cells are androgen-dependent with a low migratory ability. When they were co-cultured with B cells, there was a significant increase in cell migration of LNCaP cells, as compared to LNCaP cells alone (Fig. 1B). To test if this effect was specific to dihydrotestosterone (DHT)-responsive prostate cancer cells only, we performed a similar experiment using Du145 cells, which are DHT independent [35,36]. Furthermore, mitomycin C (MMC), a drug that inhibits cell proliferation, was used in the co-culture system of LNCaP cells (Fig. 1C), and it showed that cell proliferation had no effect on B cell-induced migration of LNCaP cells. Similarly, Du145 prostate cancer cells were also more migratory in the presence of B cells, and this B cells-enhanced cell migration of Du145 cells was not affected by MMC treatment at all (Fig. 1D and Supplemental Fig. S1). These findings suggest that B cells are critical to prostate cancer progression through enhancing cancer cell migration regardless of their responsiveness to DHT and/or their status of androgen receptors. In this current study, we focused on understanding the effect of infiltrating B cells on early-stage prostate cancer that is still responsive to DHT; therefore, LNCaP cells were used for further dissecting the underlying mechanisms.

### 3.2 Key Secretion Factors of B Cells Mediated Cell Migration of LNCaP Cells

**Figure 2:**
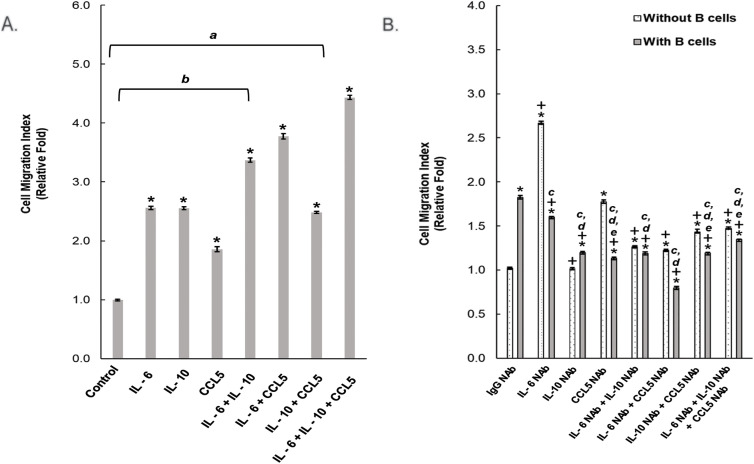
B cell-secreted cytokines promote prostate cancer cell migration. (**A**) LNCaP cells were treated with the indicated recombinant proteins in a transwell chamber to assess their migratory abilities. *: *p*-value <0.05 as compared to the control group (**B**) The indicated neutralizing antibodies (NAbs) were added to the transwell assay of LNCaP cells in the presence or absence of B cells. Figure shown is representative of three independent experiments *: *p*-value < 0.05 as compared to the control group ((**B**) IgG NAb without B cells); +: *p*-value < 0.05 as compared to IgG NAb with B cells; ANONA analysis for cell migration mean value, a: *p*-value < 0.05 as compared to IL-6 + IL-10 + CCL5; b: *p*-value < 0.05 as compared to IL-6 + CCL5 ; c: *p*-value < 0.05 as compared to IgG NAb + B cells ; d: *p*-value < 0.05 as compared to IL-6 NAb + B cells ; e: *p*-value < 0.05 as compared to IL-6 NAb + CCL5 NAb + B cells

Since cancer cell migration was observed in a Boyden chamber, where prostate cancer cells and B cells shared growth media through a porous membrane that did not allow any physical interaction, the effect of increased cancer cell migration was concluded to be resulted from the secretion factors of the B cells. Using cytokine array screening, we identified the B cell-secreted proteins in our co-culture system, as compared to LNCaP cells cultured alone. The top-ranked three most abundant secretion factors were IL-6, IL-10, and CCL5. To verify our cytokine array results, ELISA was carried out to evaluate the secreted IL-6, IL-10, and CCL5 from the collected co-culture media (Supplemental Table S1). The detected concentration of the secreted IL-6, IL-10, and CCL5 was 0.88, 2.12, and 0.59 ng/mL (Supplemental Table S1). To confirm their effect on prostate cancer cell migration, we treated LNCaP cells with human recombinant IL-6, IL-10, and CCL5 in the Boyden chamber migration assay. Our data showed that any of these three cytokines can elevate cell migration of LNCaP cells (Fig. 2A). Furthermore, MMC treatment did not change the potentiated LNCaP migratory ability in the presence of the indicated recombinant cytokine proteins, suggesting that LNCaP cell proliferation was not involved (Supplemental Fig. S2).To test if there was an additive effect among these cytokines collectively, since several cytokines could be secreted at the same time by infiltrating B cells [10,37–39], different combinations of recombinant IL-6, IL-10, and CCL5 were added to LNCaP cells for testing their abilities to regulate LNCaP cell migration. There was an additive effect in LNCaP cell migration in response to the dual treatment of IL-6 and CCL5, as compared to the elevated effects of the individual recombinant proteins (Fig. 2A). Of note, when treated with all three recombinant proteins, it had a maximal effect on LNCaP cell migration (Fig. 2A). Altogether, it suggests that these cytokines can promote cell migration individually and/or in combination among themselves.

To further substantiate our previous results, we utilized the neutralizing antibodies (NAbs) that are specific to either IL-6, IL-10, or CCL5, respectively, in the presence of B cells in the LNCaP cell migration assay. The B cells-induced cancer cell migration ability of LNCaP cells was significantly decreased when treated individually and/or with any combinations of specific neutralizing antibodies, as compared to isotype NAb control treatment (Fig. 2B). The B cell-induced LNCaP cell migration was completely abolished when depleting both IL-6 and CCL5 via a combination of IL-6 and CCL5 NAbs. Overall, these results suggest that the secreted IL-6, IL-10, and CCL5 from B cells led to the elevated migration of LNCaP cells.

### 3.3 LNCaP Underwent EMT in Response to Cytokines Secreted by B Cells Leading to Cancer Cell Migration

**Figure 3:**
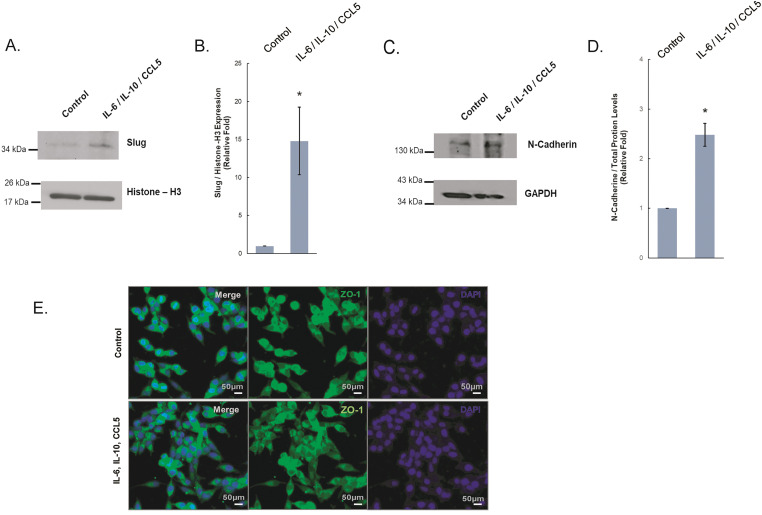

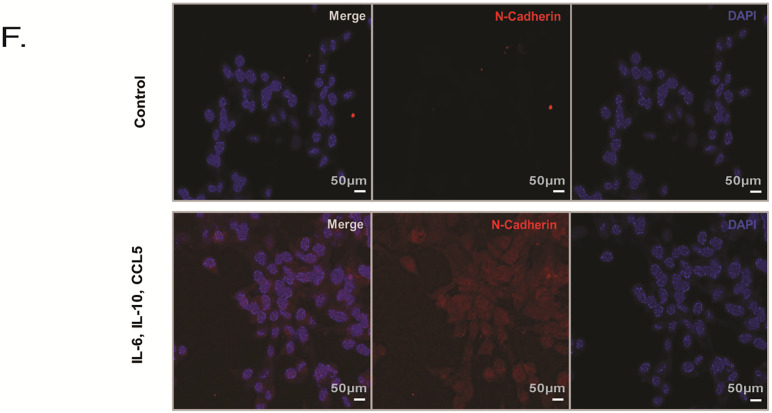
Expression of EMT proteins in LNCaP cells treated with B cell-secreted cytokines. LNCaP cells were treated with all 3 recombinant proteins including IL-6, IL-10, and CCL5. Western blot analysis was conducted for the protein expressions of (**A**) Slug, with Histone-H3, as the loading control. (**B**) Quantitative analysis of Slug expression from (**A**) was shown. (**C**) Protein expressions of N-cadherin, with GAPDH as the loading control. (**D**) Quantitative analysis of N-Cadherin expression from (**B**) was shown. (**E**,**F**) Immunocytochemistry of LNCaP cells treated with the indicated recombinant cytokines or control for expression of (**E**) ZO-1 (green), and (**F**) N-cadherin (Red) via immunofluorescence staining. Scale bar: 50 µm. Figure shown is representative of three independent experiments *: *p*-value < 0.05 as compared to the control group

In observing the effect of IL-6, IL-10, and CCL5 on prostate cancer cell migration of LNCaP, we tested if they underwent EMT, an essential process to increase cell mobility via losing epithelial cell-cell contacts. The markers for EMT include loss of E-cadherin expression, increase of N-Cadherin expression, disorganization of cell junction proteins such as ZO-1, and upregulation of transcription factors such as Slug [40–43]. We detected an upregulation of N-cadherin and Slug in LNCaP cells treated with these three combined recombinant proteins as compared to the control group (Fig. 3A–D,F). We also observed that ZO-1 was disorganized within the cell membrane of LNCaP in response to the treatment (Fig. 3E). Altogether, these results suggest that in response to IL-6, IL-10, and CCL5, LNCaP prostate cancer cells undergo EMT that leads to their increased cell migration.

## 4 Discussion

Understanding how prostate cancer cells progress in response to different components of the TME is vital to discovering new treatments for prostate cancer. Animal studies that focused on the effects of B cells within the TME have shown a decrease in tumor growth in mice that lack B lymphocytes in comparison with mice with an intact immune system [13,17,23,38]. Although these studies also implicated a role of B cells in promoting tumor growth *in vivo*, they failed to provide precise mechanisms. Our *in vitro* system allows us to manipulate each component, and we are able to reveal not only the effect of infiltrating B cells on prostate cancer cell migration but also how they achieve this. Furthermore, our system can also provide greater insight into the overall effects of infiltrating B cells at distinct stages of prostate cancer. Our results indicated a pro-tumorigenic role of infiltrating B cells in different stages of prostate cancer by increasing cancer cell migratory abilities, which is one of the hallmarks of cancer progression. The advance in the cell’s migratory aptitude promotes the cancer cell ability to metastasize in the body.

Within the tumor microenvironment, B cells can suppress T-cell activation directly or indirectly, leading to cancer progression [21,26,44]. Directly, B cells can alter the function of T cells by cell-to-cell interaction. Indirectly, B cell-secreted cytokines within the TME can block the activation of T cells as well as other types of immune cells [11,44–46]. In this study, we identified secreted factors of B cells and evaluated their effects on prostate cancer cell migration. The identified secreted factors of B cells include IL-6 [46,47], IL-10 [17,48], and CCL5 [49,50], and each individual cytokine has been linked to cancer growth through regulating inflammation within the TME [21,24,27,51]. We also demonstrated that IL-6, IL-10, and CCL5, individually or combined, can aid in the increased cell migration of LNCaP cells, thus suggesting that these secretion factors promote the ability of prostate cancer cells to move around within the prostate at early- and mid-stages of the disease, which could later lead to metastasis.

Using neutralizing antibodies targeting each specific cytokine identified in our co-culture system, we showed that LNCaP cell migration decreased in the transwell chamber when treated with these specific neutralizing antibodies, as compared to the control (Fig. 2B). There was an additive effect in prostate cancer cell migration when a dual treatment that had recombinant IL-6 and CCL5 was given. Furthermore, when both NAbs of IL-6 and CCL5 were added to the migration assay, it completely diminished the B cell-stimulated cell migration of LNCaP prostate cancer cells. The data also showed that when we treated LNCaP cells alone with the NAbs of IL-6 or CCL5, individually and/or collectively, there was an increase in migration of LNCaP cells, as compared to the control/isotype IgG NAb, which suggests that there was an intrinsic effect occurring due to the neutralization of IL-6 or CCL5 on the cell migration. However, the significant decrease in LNCaP cell migration with NAb of IL-6 and/or CCL5 in the transwell with B cells indicates that IL-6 and CCL5 have a noteworthy impact on migration (Fig. 2). This suggests a novel approach to treatments that regulates overly secreted cytokines of B cells within the TME, leading to the reduction of prostate cancer cell’s ability to metastasize.

**Figure 4:**
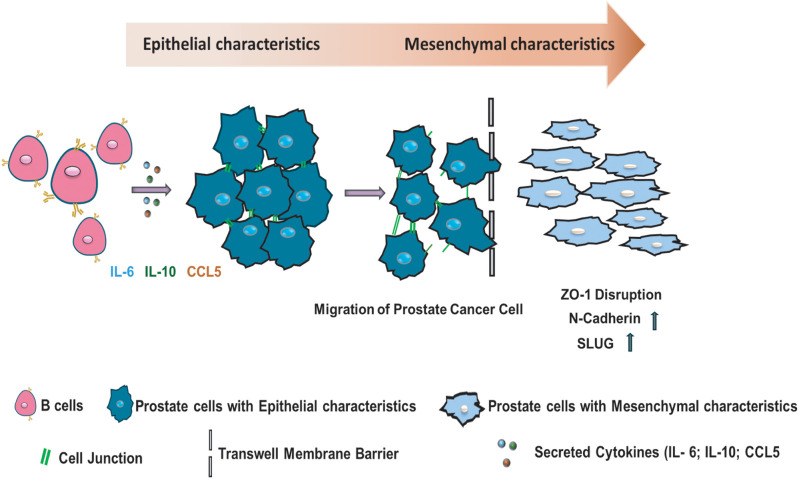
Mechanism of the infiltrating B cells regulating prostate cancer cell migration. A diagram illustrates the mechanism supported by our results, shown that B cell secretion factors: IL-6, IL-10, and CCL5 increase prostate cancer cell migration by increasing protein expression of N-cadherin and Slug, as well as the disorganization of ZO-1

Furthermore, the increased expression of N-cadherin and Slug in the prostate cancer cells that were treated with all three recombinant cytokines (Fig. 3A,B) shows that the elevated prostate cancer migration resulted from EMT (Fig. 4). Previous studies have shown that regulating specific markers, such as Slug, could lead to metastasis of prostate cancer due to suppression of cell-cell adhesion [52,53]. Based on our results shown here, these identified secretion factors could be used as a biomarker panel to monitor progression of prostate cancer to progress to a later stage. Therapeutic treatments that target the identified molecules, such as B cell secreted factors and/or EMT, could be useful in the future for controlling the advancement of prostate cancer development.

Limitation of this study is using LNCaP cells as a model for early-stage prostate cancer given that LNCaP cell line was established from the prostate cell cells metastasized in the lymph nodes despite LNCaP are androgen-dependent and -responsive. An ideal model of early-stage prostate cancer would be prostate cancer cells that are originated from the human prostate, non-metastasized cancer cells, however, not such cell lines exist so far. The other limitation is the lack of an *in vivo* model to further support our findings.

## 5 Conclusion

The secreted IL-6, IL-10, and CCL5 of infiltrating B cells enhance the cell migration of prostate cancer cells, through inducing EMT. Depletion of these B cell-secreted cytokines via their specific neutralizing antibodies inhibits the migration of LNCaP prostate cancer cells induced by infiltrating B cells. Collectively, IL-6, IL-10, and CCL5 could be used as biomarkers to evaluate and monitor early stages of prostate cancer progression.

## Supplementary Materials









## Data Availability

The data generated in this study are available within the article and Supplementary Materials.

## Supplementary Materials

The supplementary material is available online at https://www.techscience.com/doi/10.32604/or.2025.073532/s1.

## Abbreviations

CCL5: C-C motif chemokine ligand 5
EMT: epithelial-mesenchymal transition
FBS: fetal bovine serum
IgG: immunoglobulin G
IL-6: interleukin-6
IL-10: interleukin-10
NAbs: neutralizing antibodies
TME: tumor microenvironment
ZO-1: Zonula Occludens-1
MMC: mitomycin C
