# Supplementary figures and images for: Cytokines IL-6, IL-10, and CCL5 Secreted by Infiltrating B Cells Promote Cell Migration of Human Prostate Cancer Cell Lines
Source: Oncol Res. 2026 Mar 23;34(4):15. doi: 10.32604/or.2025.073532 (PMC13040286; doi:10.32604/or.2025.073532)

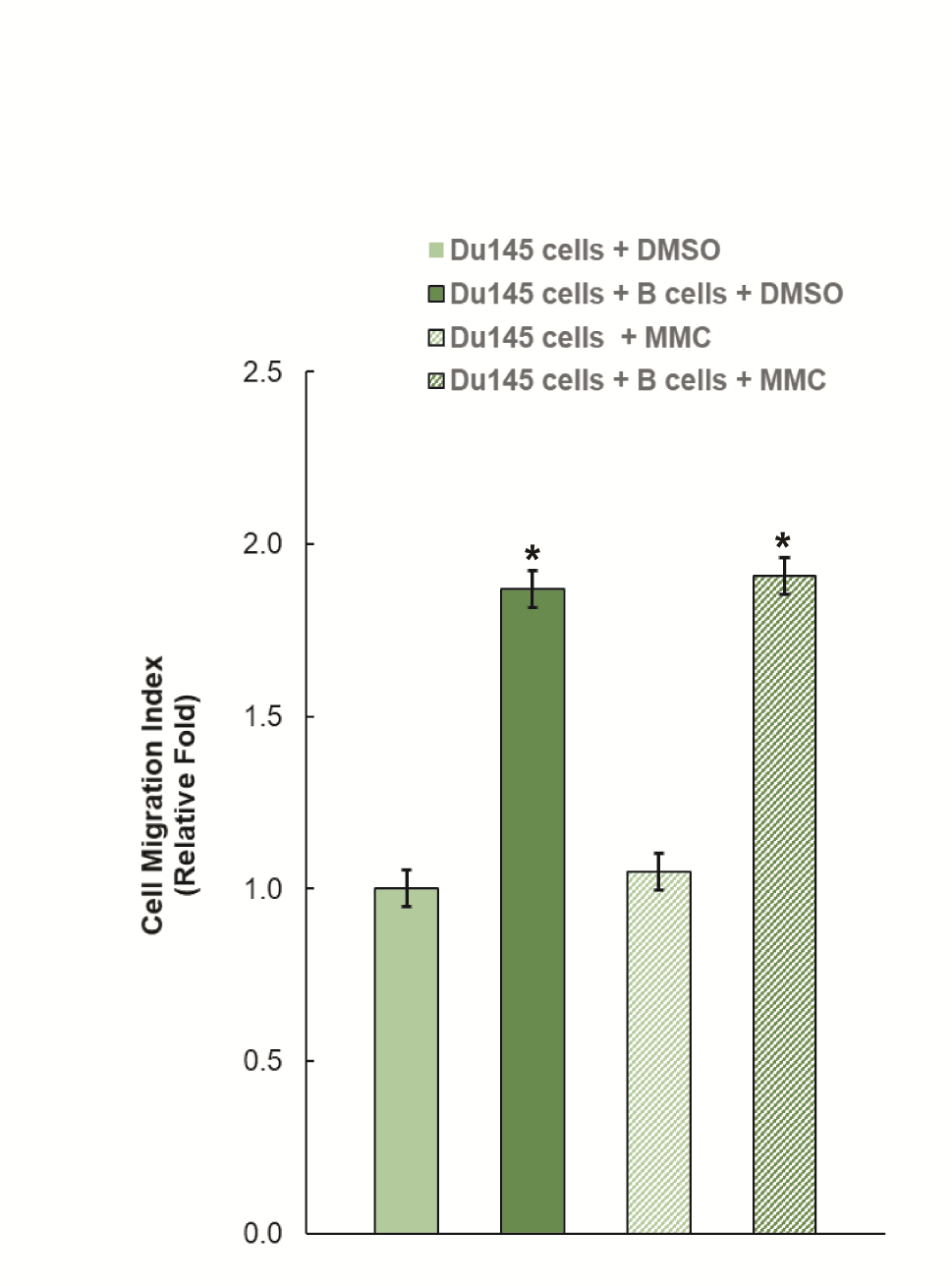

Supplement: Supplementary file 1 [file OncolRes-34-73532-s001.tif]

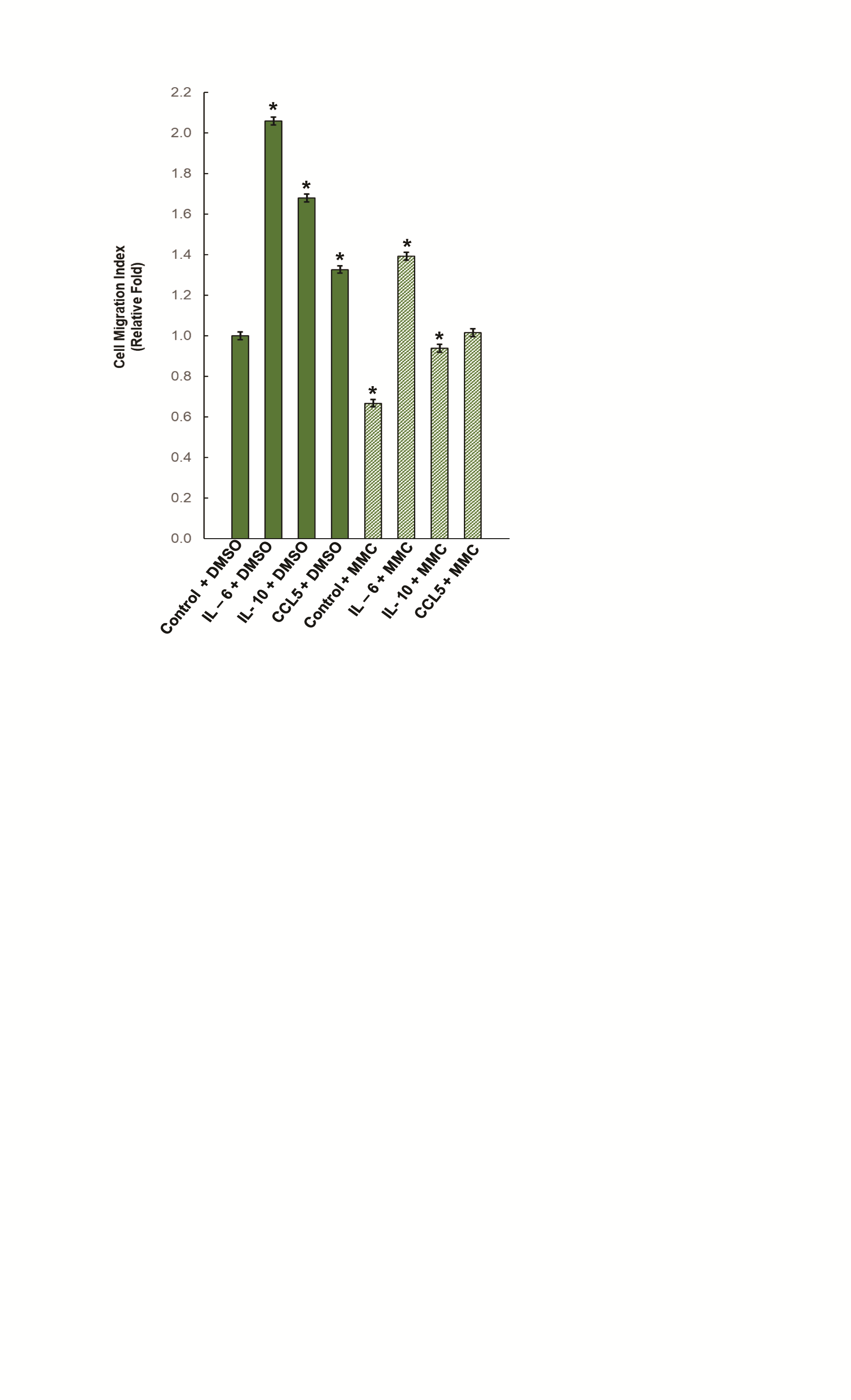

Supplement: Supplementary file 2 [file OncolRes-34-73532-s002.tif]
