# Supplementary material for: Cytokines IL-6, IL-10, and CCL5 Secreted by Infiltrating B Cells Promote Cell Migration of Human Prostate Cancer Cell Lines
Source: Oncol Res. 2026 Mar 23;34(4):15. doi: 10.32604/or.2025.073532 (PMC13040286; doi:10.32604/or.2025.073532)
Supplement: Supplementary file 3 [file OncolRes-34-73532-s003.docx]

Supplementary Materials and Methods


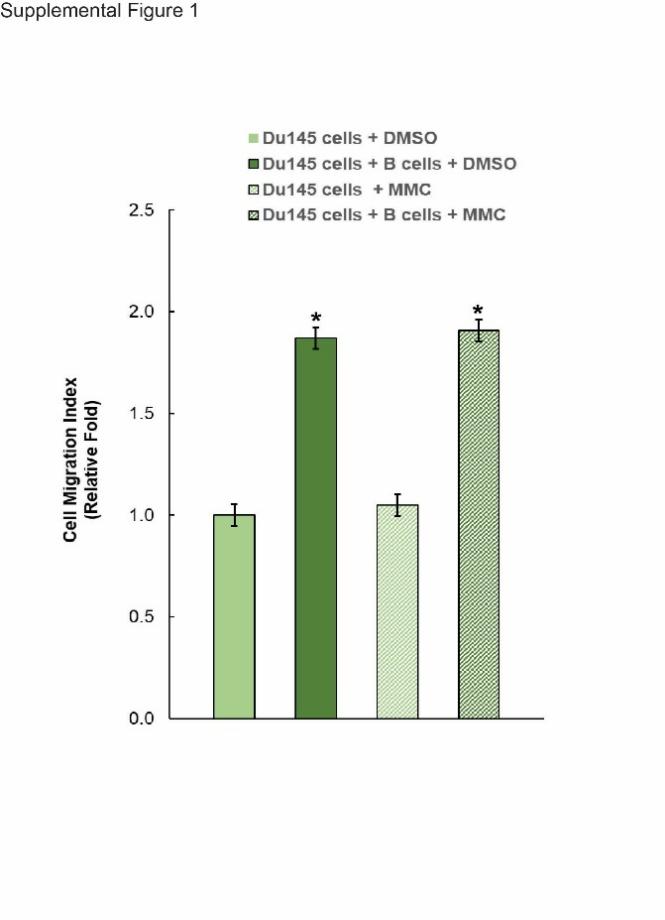


**Supplemental Figure 1: MMC effect Du145 cell migration co-cultured with B cells.** Treatment of mitomycin C (MMC) in the co-culture system for Du145 cells that were cultured with B cells. For the treatment dose and duration, see Section 2.2 of Materials and Methods. *: *p*-value < 0.05 as compared to the DMSO-treated prostate cancer cells that were cultured alone (control).


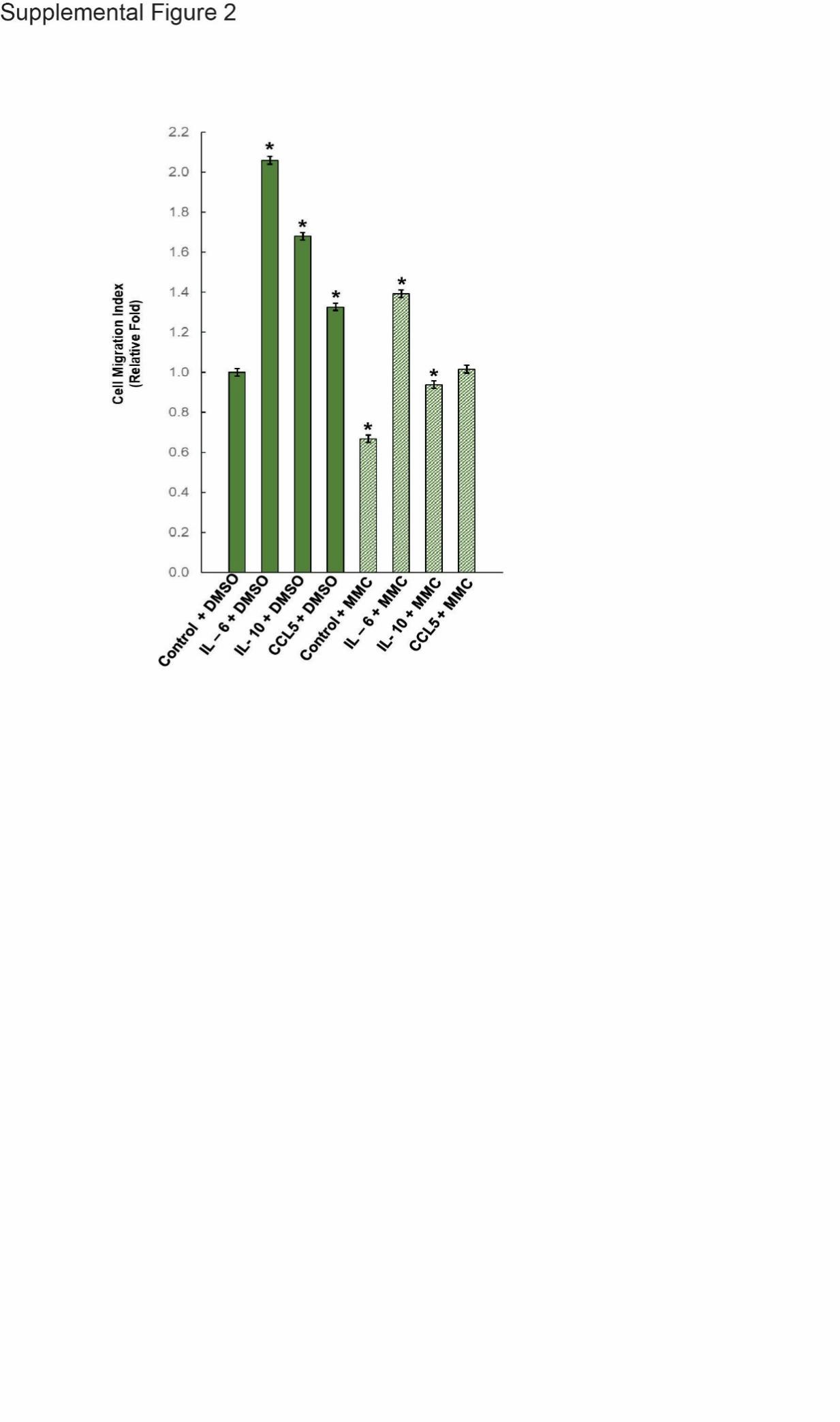


**Supplemental Figure 2: Effect of MMC on cell migration of LNCaP cell treated with recombinant protein IL-6, IL-10, or CCL5.** LNCaP cells were treated with the indicated recombinant protein IL-6, IL-10 or CCL5 in the presence or absence of mitomycin C (MMC) for evaluating their migratory abilities in Transwell^®^ assays. For the treatment dose and duration, see Section 2.2 in the Materials and Methods. *: *p*-value < 0.05 as compared to the control/DMSO group.

**S1 Enzyme-linked immunosorbent assay (ELISA)**

Human ELISA kits were used for detecting levels of secreted IL-6, IL-10, and CCL5. ELISA kits for human IL-10 (Cat. # RAB0244) and human RANTES/CCL5 (Cat. # RAB007) were purchased from Sigma (St. Louis, Missouri, USA). Human IL-6 ELISA kit (Cat. # 501030) was purchased from Cayman Chemical Company (Ann Arbor, Michigan, USA). The assays were carried out according to the manufacturer’s protocols using Agilent BioTek Synergy Neo2 Hybrid Multi-Mode Reader (Agilent Technologies, Inc., Santa Clara, California, USA).

**Supplemental Table 1Detected levels of cytokines secreted in the culture media.**

| **Secretion Factors/Conditions** | **LNCaP cells only** | **LNCaP cells and B cells** |
| --- | --- | --- |
| Interleukin-6 (IL-6) | 0.04 ng/mL | 0.88 ng/mL |
| Interleukin-10 (IL-10) | 0.00 ng/mL | 2.12 ng/mL |
| RANTES (CCL5) | 0.00 ng/mL | 0.59 ng/mL |
